# Multi-Dimensional Antioxidant Screening of Selected Australian Native Plants and Putative Annotation of Active Compounds

**DOI:** 10.3390/molecules28073106

**Published:** 2023-03-30

**Authors:** Md. Ahsan Ghani, Celia Barril, Danny R. Bedgood, Geoffrey E. Burrows, Danielle Ryan, Paul D. Prenzler

**Affiliations:** 1 School of Agricultural, Environmental and Veterinary Sciences, Charles Sturt University, Wagga Wagga, NSW 2650, Australia; gahsan23@yahoo.com (M.A.G.); cbarril@csu.edu.au (C.B.); dbedgood@csu.edu.au (D.R.B.J.); gburrows@csu.edu.au (G.E.B.); dryan@csu.edu.au (D.R.); 2 The Gulbali Institute, Charles Sturt University, Wagga Wagga, NSW 2650, Australia

**Keywords:** total phenolic content, lipid oxidation, natural products

## Abstract

*Acacia implexa, Eucalyptus rossii* and *Exocarpos cupressiformis* are native plants of Australia, which were used by the First Peoples for medicinal purposes. In this study, 70% aqueous ethanol crude extracts were prepared from *A. implexa* bark and leaves, *E. rossii* leaves and *E. cupressiformis* leaves, and partitioned via sequential extraction with *n*-hexane, dichloromethane (DCM), ethyl acetate and ethanol. The crude extracts and fractions were screened for antioxidant activity using a novel, high-throughput lipid-based antioxidant assay, as well as the aqueous ABTS (2,2′-azino-bis(3-ethylbenzothiazoline-6-sulfonic acid)) assay and the Folin–Ciocalteu test for total phenols. In the lipid-based assay, non-polar *n*-hexane and DCM fractions showed higher antioxidant activity against the formation of peroxides and thiobarbituric acid reactive substances (TBARS) than the other fractions, whereas the non-polar fractions were not effective in aqueous assays. This illustrates that the high potential of the lipid-soluble *n*-hexane and DCM fractions as antioxidants would have been missed if only aqueous-based assays were used. In addition, the potent antioxidant compounds were putatively annotated using liquid chromatography quadrupole time-of-flight mass spectrometry (LC-qTOF-MS). Gallic acid, (+)-catechin, (−)-epicatechin and tannins were found in most crude extracts.

## 1. Introduction

Lipid oxidation and antioxidant activity continue to be an active area of research [1]. Lipid oxidation has been implicated in various diseases such as arthritic, cardiovascular and neurodegenerative diseases [2], and such oxidation also causes rancidity of food resulting in quality deterioration for the food industry [3]. Oxidation occurs in the presence of heat, light, trace amounts of metal ions, molecular oxygen or reactive oxygen species—all of which can interact with lipids to generate peroxides as primary oxidation products. Lipid peroxides then undergo breakdown to form numerous secondary oxidation products [3]. Some of the secondary oxidation products are hexanal and thiobarbituric acid reactive substances (TBARS), including malondialdehyde (MDA) [1].

Natural products, such as those found in medical plant extracts, are potential antioxidants because of their capacity to limit lipid oxidation by scavenging free radicals, binding metal ions, or inhibiting the formation of the above oxidation products. Medicinal plants are broadly researched in order to find new antioxidants, and Ortego-Ramirez et al. [4] argue that the customary use of these plants over thousands of years demonstrates the potential to discover new and potent antioxidants. In addition, they are considered safer than synthetic antioxidants such as butylated hydroxyanisole (BHA) and butylated hydroxytoluene (BHT), and there is an enhanced customer preference toward natural antioxidants.

Screening plant extracts for antioxidant activity typically involves the use of several in vitro assays, such as the Folin–Ciocalteu test for total phenols, anti-radical scavenging activity using, e.g., 2,2′-azino-bis(3-ethyl-benzothiazoline-6-sulfonic acid) (ABTS^●+^), and the ferric reducing antioxidant power (FRAP) assay [5]. These tests are mostly conducted in aqueous solution and may not adequately reveal antioxidants that are effective in lipid systems. On the other hand, a method for rapid screening of antioxidant activity in a lipid system has been recently developed [6], which has the potential to evaluate antioxidants that may be effective for use in oils, fats and emulsions. Moreover, Frankel and Meyer [7] and McDonald et al. [8] recommended the use of orthogonal assays, i.e., those that test different activities (radical scavenging, chain-breaking, etc.) and the use of both aqueous- and lipid-based antioxidant tests fulfill this recommendation.

Australia is one of 17 megadiverse countries [9], with over 19,000 native plant species described [10]. Within Australia, the state of New South Wales (NSW) is home to ~4700 native species of plants, many of which were used for food and/or medicines by the First Peoples. The Wiradjuri nation, the largest in NSW, published a compendium of plant use [11] from which three species of plants were selected for this study—*Acacia implexa*, *Eucalyptus rossii* and *Exocarpos cupressiformis*. The choice was based on four criteria: (1) readily available near Wagga Wagga; (2) reported medicinal use; (3) abundant, i.e., not endangered; and (4) having no or few studies of their phytochemistry/bioactivity. For *A. implexa*, the bark has been reported to be used to treat skin complaints and sores [12], and the authors are aware of only one report [12] on the broad phytochemical screening into classes such as alkaloids, steroids, etc., as well as total phenolic content, total flavonoid content and total condensed tannin content. The same article also reported antioxidant activity of *A. implexa* bark as measured by the ABTS, 2,2-diphenyl-1-picrylhydrazyl (DPPH) and FRAP tests. Apart from Akter et al. [12], there is only one other report on the phytochemistry of *A. implexa*, where a limited number of flavonoids were described [13]. No reports are available on the antioxidant activity of *E. rossii*, nor *E. cupressiformis*, as far as the authors are aware. Similarly, there is limited literature on the phytochemistry of these species—Saraf et al. [14] reported four flavonoids in *E. rossii*, and Cook and Haynes [15] reported four flavonoid glycosides in *E. cupressiformis*.

The objective of this study was to screen extracts of *A. implexa, E. rossii* and *E. cupressiformis* for antioxidant activity and identify/putatively annotate potentially active antioxidant compounds. In particular, we were interested in ranking antioxidants using a variety of antioxidant assays so that highly active extracts could be identified for further studies in food or medicinal applications.

## 2. Results and Discussion

### 2.1. Extraction Yield

Percentage yields of crude extracts are shown in Table 1. For *A. implexa*, the yield for leaves (28%) was higher than for bark (11%). The yields from the leaves of *E. rossii* and *E. cupressiformis* were similar to those of *A. implexa* leaves, i.e., 30% and 28%, respectively. The results for fractions (Table 1) show that EtOAc and EtOH solvents generally produced the highest yields: EtOAc fractions ranged from 3 to 30%, and EtOH fractions from 0.8 to 34%. The less polar solvents DCM and *n*-hexane had generally lower yields ranging from 0.3 to 15% and 0.08 to 3%, respectively. As the crude extracts were prepared from 70% (*v*/*v*) aqueous EtOH, it is not surprising that higher extractions were observed in the more polar solvents.

### 2.2. Determination of Total Phenolic Content (TPC)

Phenolic compounds are important plant secondary metabolites [16], which contribute to the antioxidant activity of extracts, and typically total phenolic content (TPC) is measured prior to antioxidant assays. Table 1 shows that crude extracts of all species had appreciable TPC ranging from 195.2 to 439.5 mg GAE/g dry extract. Of the crude extracts, *A. implexa* bark contained the highest TPC with 439.5 mg GAE/g dry extract, followed by *E. cupressiformis* at 437.6, *E. rossii* at 419.2 and *A. implexa* leaves at 195.2 mg GAE/g dry extract. In *A. implexa* bark, the phenolic content may be due mainly to tannins. Bourais et al. [17] found higher TPC in bark than leaves in extracts of *Juglans regia* In bark, the different phenolic acids such as caffeic acid, ferulic acid and gallic acid are generally condensed over time to form polymeric substances. These are evident in the chromatograms at 280 nm (Section 3.4) as regions of broad increased intensity (the so-called polymeric “hump” [18]). The polymeric hump is larger for AIB than AIL.

The antioxidant activity of various Australian native plants has been recently reviewed [19], including various *Acacia* and *Eucalyptus* species; however, as far as the authors are aware, there is only one report in the literature on the antioxidant activity of *A*. *implexa* [12], which showed that a crude extract of the bark was high in TPC (with a value similar to this work) and ABTS radical scavenging activity. The leaves were not included in that study, nor was the crude extract fractionated. No studies were found on the antioxidant activity of *E. rossii* or *E. cupressiformis* extracts from any plant parts.

When the extracts were fractionated with *n*-hexane, DCM, EtOAc and EtOH, TPC varied depending on the solvent polarity. The TPC of EtOAc fractions ranged from 328.5 to 583.2 mg GAE/g dry extract, followed by EtOH from 118.7 to 439.7 mg GAE/g dry extract. The DCM fractions ranged from 98.6 to 185.8 mg GAE/g dry extract, followed by *n*-hexane fractions from 42.4 to 106.4 mg GAE/g dry extract. The results demonstrate that EtOAc fractions possessed the highest TPC, while the least polar *n*-hexane had the lowest values. These results of TPC for EtOAc and *n*-hexane fractions were consistent with those for *Acacia confusa* [20] and *Cinnamomum osmophloeum* [21].

### 2.3. Screening Antioxidant Activity

#### 2.3.1. 2,2′-Azino-bis(3-ethylbenzothiazoline-6-sulfonic acid) (ABTS) Assay

**Figure 1:**
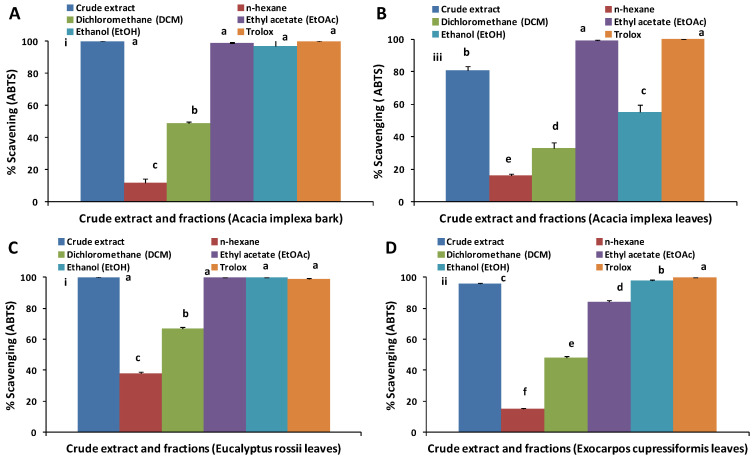
Radical scavenging activity for crude extract and fractions of: (**A**) *A. implexa* bark, (**B**) *A. implexa* leaves, (**C**) *E. rossii* leaves and (**D**) *E. cupressiformis* leaves. Different letters (a–f) in crude extract and fractions of same species show significant difference at *p* < 0.05. Different small Roman numerals (i–iii) show significant difference at *p* < 0.05 for crude extracts of different species. Results are presented as mean ± standard deviation (n = 3), and statistically significant difference was determined by one-way ANOVA and Tukey’s HSD post hoc test.

The free radical scavenging capability of the crude extracts and fractions were investigated by the ABTS assay. Figure 1 illustrates that all crude extracts and fractions of all species showed varying amounts of free radical scavenging activity. Crude extracts of all species showed a high percentage of radical scavenging ranging from 81% to 100%: AIB (100%) = EUR (100%) > EXOC (96%) > AIL (81%). This correlates well (*r*^2^ = 0.94) with the TPC of the crude extracts of *A. implexa* bark and *E. rossii* (Table 1).

The EtOAc and EtOH fractions showed the highest activities (55–100%), while the lowest radical scavenging activity was found among the *n*-hexane fractions (12–38%), which reflects similar trends in the TPC data. Barapatre et al. [22] and Tung et al. [20] also found both high TPC and ABTS radical scavenging activity in the EtOAc fractions of *A. nilotica* and *A. confusa*, respectively. In studies where a crude extract was fractionated by solvents of different polarities, it is often found that higher antioxidant activity occurs in the EtOAc fraction than in *n*-hexane or DCM fractions [23,24], consistent with the findings of this study.

The low TPC and antioxidant activity of *n*-hexane and DCM fractions can be attributed to the fact that, being non-polar, the fractions may possess mostly lipophilic compounds, which typically do not show strong activity in aqueous assays. At this stage of a screening study, the focus usually turns to extracts/fractions that have shown activity in these aqueous assays, yet, as we have highlighted [1], this may result in non-polar fractions being overlooked, when in fact they may show significant activity in non-aqueous assays. Therefore, antioxidant activities of all the crude extracts and fractions were assessed with the lipid-based FTC and TBARS assays.

#### 2.3.2. Ferric Thiocyanate (FTC) Assay

**Figure 2:**
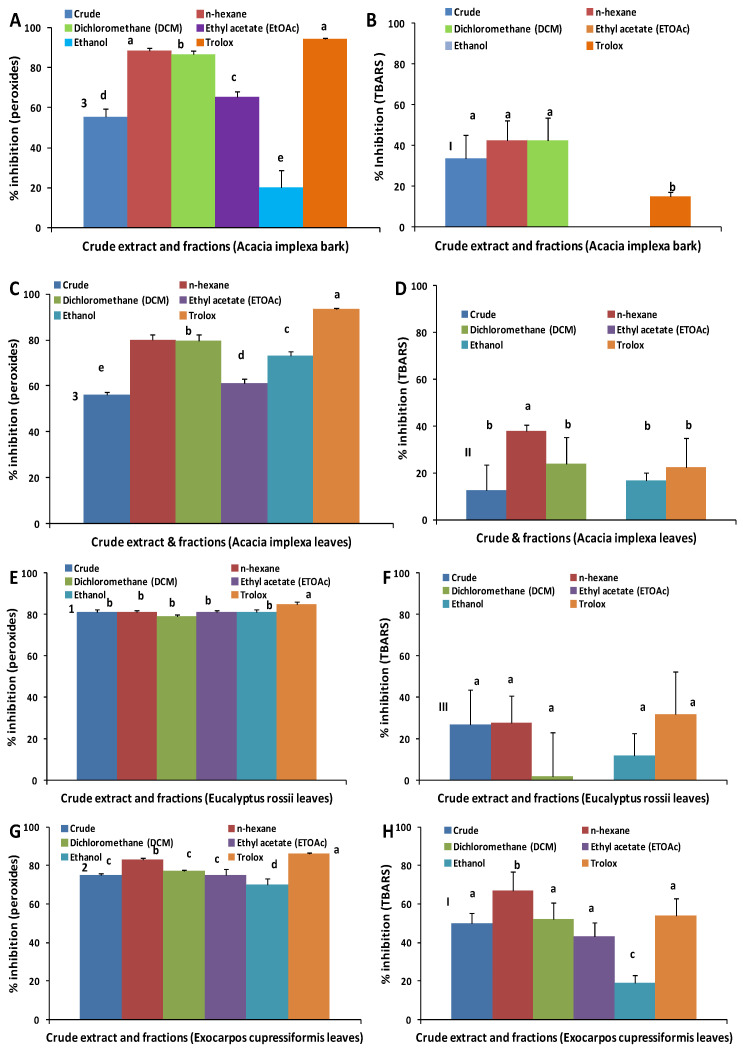
Antioxidant activity (% inhibition) for the FTC (peroxides) assay: (**A**) *A. implexa* bark, (**C**) *A*. *implexa* leaves, (**E**) *E. rossii* leaves and (**G**) *E. cupressiformis* leaves; and for the TBARS assay: (**B**) *A. implexa* bark, (**D**) *A. implexa* leaves, (**F**) *E. rossii* leaves and (**H**) *E. cupressiformis* leaves. Different letters (a–e) in crude extract and fractions of same species show significant difference; different numbers (1–3) for FTC and different capital Roman numerals (I–III) in crude extracts of the different species show significant difference at *p* < 0.05. Results are presented as mean ± standard deviation (n = 3), and statistically significant difference was determined by one-way ANOVA and Tukey’s HSD post hoc test.

Figure 2A,C,E,G shows that the crude extracts of all species had appreciable activity in preventing peroxide formation. Percent inhibition for the crude extracts was EUR (81%) > EXOC (75%) > AIL (56%) = AIB (55%), with statistically significant differences (*p* < 0.05) among all extracts except AIL and AIB. These results may be compared with the radical scavenging activity shown in Figure 1; in both cases, EUR and EXOC had the highest and second highest activities, respectively, and AIL had the lowest activities. In contrast, AIB had the highest (equal to EUR) radical scavenging activity, but the equal (to AIL) lowest percent inhibition of peroxides. Sultana et al. [25] also reported similar contrasting results for a crude extract of bark from *Acacia nilotica*, with high activity using the FTC assay. As will be seen below, when fractions are discussed, it is possible to have contrasting results in aqueous versus lipid-based assays. It is well known that the chemistry in a multi-component lipid emulsion system is much more complex than a relatively simple aqueous system (solvent and ABTS^+•^), and as such, antioxidant activity can be influenced by diffusion, and interfacial and other phenomena, which are not present in an aqueous system [3]. However, if antioxidant extracts and fractions are to be applied in a lipid-based system, then it is important to undertake testing in such a system, as this study shows the results are not always congruent.

Figure 2C shows that the crude fraction of AIL had the lowest peroxide inhibition activity, compared with the fractions. A similar result is found for AIB (Figure 2A), although here the crude fraction is only lower in activity than the *n*-hexane, DCM and EtOAc fractions. Singh et al. [26] have also reported this phenomenon—that fractions were comparatively more effective antioxidants than the crude extract in *Acacia auriculiformis*. One possible explanation [27] is that the compounds present in the crude extract may exert antagonistic effects that might provide lower net activity. In fractions, these compounds may be removed, resulting in higher overall activity.

The most striking result shown in Figure 2 is that the *n*-hexane and DCM fractions consistently showed high activity for peroxide inhibition compared to their radical scavenging activity (Figure 1). This is true across fractions from all species, but AIB and AIL fractions are most notable in that peroxide inhibition for the *n*-hexane and DCM fractions are higher than for the EtOAc and EtOH fractions (*p* < 0.05), in complete contrast to the corresponding results for radical scavenging activity (*p* < 0.05). As highlighted above, this reversal in antioxidant activity between lipid and aqueous systems has important implications for screening studies such as this. As reviewed by Ghani et al. [1], it is not uncommon for aqueous-based antioxidant assays to be used as primary screens, with only the highest activity extracts or fractions tested in subsequent lipid-based assays. If this approach were taken here, the *n*-hexane and DCM fractions of AIB and AIL would not have been included in the FTC assay and the significant activity of these fractions would not have been discovered.

Overall, the *n*-hexane fraction of AIB showed the highest antioxidant activity (89%), followed by the DCM fraction of AIB (87%). The high activity of the *n*-hexane fraction of *Sedum sarmentosum* in inhibiting peroxide formation, yet with low antioxidant activity in aqueous assays, has been reported by Mo et al. [28]. Zin et al. [27] have suggested that non-polar phenolic or other compounds with diverse molecular structures may display antioxidant activity in a lipid-based system, but can be relatively inactive in aqueous systems, which tend to emphasise the antioxidant activity of highly polar phenolic compounds.

#### 2.3.3. Thiobarbituric Acid Reactive Substances (TBARS) Assay

TBARS are secondary oxidation products that have been associated with off-flavours in meat [29], as well as being potentially toxic [30]. Thus, it is important to test antioxidants for prevention of formation of TBARS, just as it is important to test for inhibition of the primary oxidation products, peroxides. All samples tested show less effective inhibition of TBARS formation compared to peroxide formation, consistent with the report of Mo et al. [28]. The crude extracts ranked in order of activity as EXOC (50%) ≈ AIB (33%) > EUR (26%) > AIL (13%) (Figure 2H,B,F,D, respectively). The order of activity is somewhat different to that for the FTC assay where EUR showed the highest activity (Figure 2E) in the FTC assay, but the second lowest activity in the TBARS test, and AIB showed the lowest activity in the FTC assay (Figure 2A), but equal highest activity in the TBARS test. Thus, it may be concluded that the components in the crude extracts that are effective in limiting peroxide formation may not be the same compounds that inhibit TBARS formation. By way of contrast, other research has found a close correspondence between inhibition of peroxides and TBARS [31,32]. This once again highlights the advantages of conducting multiple antioxidant tests to determine which samples are effective in their activities against different oxidation products.

The fractions showed more variation in their inhibition of TBARS than inhibition of peroxides. This is particularly evident in the zero activity of AIB EtOAc, AIB EtOH, AIL EtOAc and EUR EtOAc (Figure 2B,D,F). All of these showed some inhibition of peroxides, and most inhibited more than 50% peroxide formation (the exception was AIB EtOH at 20%). The difference in activity between the two types of oxidation products is further evidence of the different suites of compounds in the fractions being more or less effective antioxidants depending on whether peroxides or TBARS were measured. This observation for the fractions parallels that for the crude extracts above. A study on the antioxidant activity of Maillard reaction products in an emulsion system [33] also found that some fractions were more or less effective in inhibiting peroxides compared to TBARS.

The results for the *n*-hexane and DCM fractions for TBARS inhibition mostly match the results for peroxide inhibition. That is, the more lipophilic compounds in these fractions are more effective than the polar compounds in the EtOAc and EtOH fractions at inhibiting TBARS in the lipid system (especially shown in Figure 2B,D). The EtOAc fraction for EXOC is notable in that it is the only EtOAc fraction among the species that shows antioxidant activity in the TBARS assay (Figure 2H). In fact, the fractions of EXOC are notable in that all fractions show some antioxidant activity, whereas for the other species, one or more fractions show no activity.

Based on the overall screening of antioxidant activity of the extracts and fractions, it can be noted that antioxidant activity of crude extracts and polar fractions could be effectively measured by the ABTS assay whereas this assay could not effectively determine the activity of the non-polar *n*-hexane and DCM fractions. The activity of these fractions could be very effectively screened by the lipid-based FTC and TBARS assays. In order to further understand the possible cause of these different activities in aqueous and lipid systems, ultra-high-performance liquid chromatography diode array detection quadrupole time-of-flight mass spectrometry (UHPLC-DAD-qTOF-MS) was undertaken to provide putative annotation [34] of compounds.

### 2.4. UHPLC-DAD-qTOF-MS

**Figure 3:**
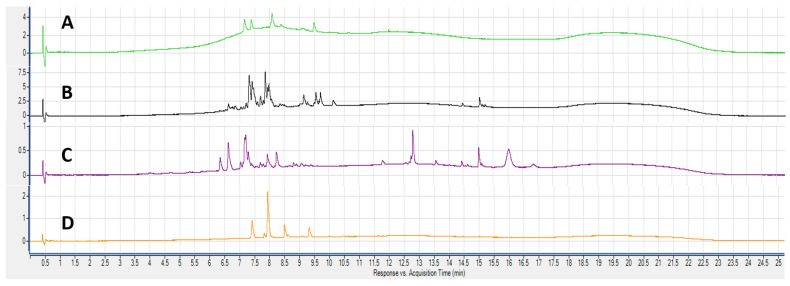
Reverse-phase chromatograms at 380 nm for crude extracts of: (**A**) *A. implexa* bark; (**B**) *A. implexa* leaves; (**C**) *E. rossii* leaves; and (**D**) *E. cupressiformis* leaves.

**Figure 4:**
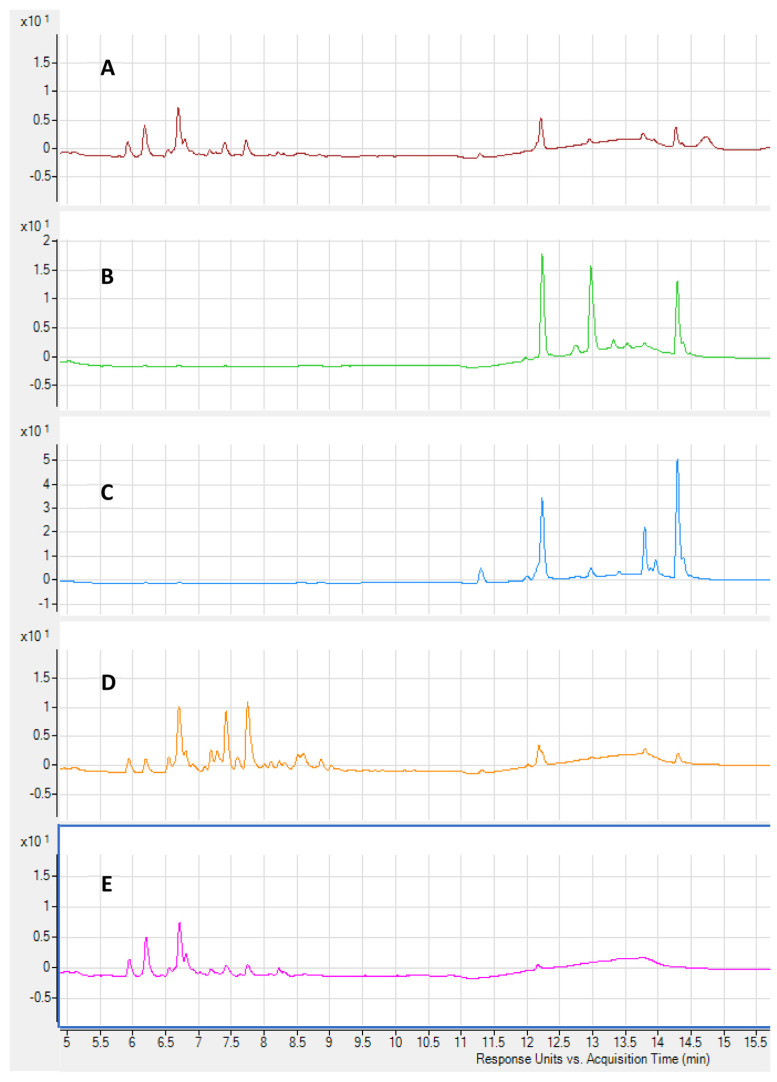
Reverse-phase chromatograms at 380 nm for the crude extract and fractions of *E. rossii*. (**A**) crude extract; (**B**) *n*-hexane; (**C**) dichloromethane; (**D**) ethyl acetate; and (**E**) ethanol.

**Figure 5:**
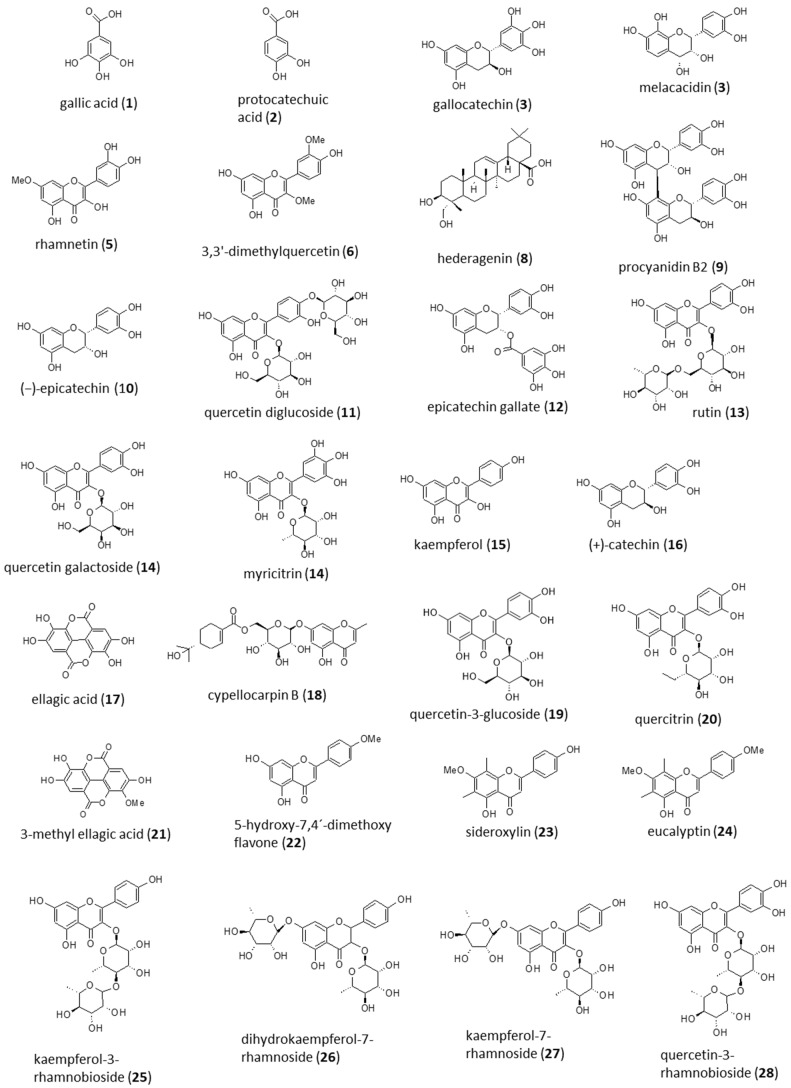
Structures of compounds putatively annotated in crude extracts and fractions of *A. implexa* bark, *A. implexa* leaves, *E. rossii* leaves and *E. cupressiformis* leaves. (Figure prepared with ACD/ChemSketch (Freeware) 2 January 2021).

Qualitative analyses of *A. implexa* bark and leaves, and *E. rossii* and *E. cupressiformis* leaves were conducted based on the major peaks found at 280 nm (general polyphenols) and 380 nm (flavonoids) using DAD, and in total ion chromatograms (TICs) from ESI-qTOF-MS. In total, 60 chromatograms were generated, 12 from crude extracts and 48 from fractions, and selected chromatograms are shown in Figure 3 and Figure 4 along with putatively annotated compounds in Table 2, Table 3, Table 4 and Table 5. The major peaks of the 70% (*v*/*v*) aqueous ethanol crude extracts were analysed first, followed by fractions. Putative annotation of the major peaks acquired at 280 nm and 380 nm was attempted by using extracted ion chromatograms (EICs) at the *m*/*z* value obtained from TICs with retention time (RT) corresponding to peaks from the DAD. The emphasis was on attempting to identify compounds likely to display antioxidant activity (polyphenols and flavonoids) in the various tests above, and beginning to link activity with possible compounds. Gallic acid, (+)-catechin and (−)-epicatechin, were identified using standards. The other 25 compounds were putatively annotated based on the literature and spectrometric data (Figure 5).

Figure 3 shows the chromatograms obtained at 380 nm from the crude extracts. A number of trends can be observed that are also evident in the chromatogram with 280 nm detection and the TICs. AIB is characterised by a broad area of intensity (3–15 min, Figure 3A), which we have previously [18] ascribed to polymeric material, presumably tannins. The prominence of this feature for AIB is consistent with many reports in the literature of the high levels of tannins found in bark [35,36]. Apart from this, each chromatogram was unique in the number and intensity of peaks, suggesting unique suites of flavonoids. AIB and EXOC have a few discernable peaks (Figure 3A,D), whereas AIL and EUR have considerably more (Figure 3B,C). Although the chromatogram for AIL shows a relatively rich abundance of compounds (Figure 3B), the crude extract of AIL had the lowest TPC. It is possible that the compounds present in the crude extract, although flavonoids, may be of a type that return a low value for the TPC test. This is usually attributed to the lack of an ortho-diphenol unit in the flavonoid structure [37]. The TPC for AIB is likely attributed to the tannins, whereas for EXOC (Figure 3D), the one major peak (quercitrin, see below) may be the main source of total phenols in the TPC test.

Figure 4 shows chromatograms generated at 380 nm for EUR crude extract and all fractions. The *n*-hexane and DCM fractions show peaks at later elution times, and hence contain less polar compounds. This is consistent with these hydrophobic solvents preferentially extracting non-polar compounds from the crude extracts. Conversely, the EtOAc and EtOH chromatograms show earlier eluting peaks due to more polar compounds, consistent with these solvents being more polar than *n*-hexane and DCM.

As mentioned in the Introduction, very few studies have been reported on the phytochemistry of the species studied here. In addition to the references cited in the introduction, the phytochemistry of other related species, e.g., Eucalyptus [38], have been used to putatively annotate peaks in the chromatograms of crude extracts and fractions.

#### 2.4.1. *Acacia implexa* Bark

In Table 2, compound **1** (see Figure 5 for structure) was assigned as gallic acid. The retention time and *m*/*z* matched those of a gallic acid standard (RT = 0.713 min and *m*/*z* = 169.0149). Assignment of gallic acid was also considered on the basis of literature for other *Acacia* species [39,40]. Compound **2** was assigned as protocatechuic acid, which was reported in *Acacia nilotica* [40]. Compounds **3**–**7** were assigned as gallocatechin/melacacidin, an isomer of gallocatechin/melacacidin, rhamnetin, 3,3′-dimethyquercetin and an isomer of rhamnetin, respectively, which were reported in either *A. implexa* [13] or other *Acacia* species [41]. Compound **8** was assigned as hederagenin, which is a plant triterpenoid. *Acacia* species are known to contain saponins [42], but as of yet, none have been reported in *A. implexa* as far as the authors are aware.

Compounds **1**–**4** were prominent in the crude extract and EtOAc fraction, and less so in the EtOH fraction, consistent with these compounds being early eluting and hence more polar. The presence of the polar compounds in the above fractions and crude extracts might contribute to the high antioxidant activity in aqueous-based ABTS assay (Figure 1A), and modest antioxidant activity in lipid-based FTC assay (Figure 2A). Previous studies [43,44] have shown that gallic acid **1** and derivatives **3**, **4** have strong antioxidant activity in the ABTS test. Compounds **5**–**8**, being less polar in nature, were prominent in *n*-hexane or DCM fractions, and hence likely contributed to the high antioxidant activity in both FTC and TBARS assays (Figure 2A,B). In particular, compounds **5** and **7** are flavonoids containing an ortho-diphenol group, which is known to convey significant activity in antioxidant assays [6].

**Table 2:** Tentative assignment of compounds in *Acacia implexa* bark (crude extract).

* Comp	* RT	* *m*/*z* Expt.	* *m*/*z* Calc	* MF	* δ in ppm	Putatively Identified Compound	Reference
1	0.714	169.0146	169.0142	C_7_H_6_O_5_	2.08	gallic acid	* ST
2	1.014	153.0192	153.0930	C_7_H_6_O_4_	0.86	protocatechuic acid	[40]
3	1.147	305.0671	305.0667	C_15_H_14_O_7_	0.08	gallocatechin/melacacidin	[13,41]
4	2.629	305.0670	305.0667	C_15_H_14_O_7_	1.06	isomer of gallocatechin/melacacidin	[13,41]
5	7.418	315.0512	315.0510	C_16_H_12_O_7_	0.55	rhamnetin	[41]
6	8.117	329.0665	329.0667	C_17_H_14_O_7_	0.53	3,3′-dimethyl-quercetin	[41]
7	9.524	315.0518	315.0510	C_16_H_12_O_7_	2.45	isomer of rhamnetin	[41]
8	12.980	471.3483	471.3480	C_30_H_48_O_4_	0.67	hederagenin	[45]

* Comp = compound, * RT = retention time, * *m*/*z* = mass to charge ratio, * expt = experimental, * calc = calculated, * MF = molecular formula, * δ in ppm = error (ppm), * ST = standards.

#### 2.4.2. *Acacia implexa* Leaves

In Table 3, compound **4** was also assigned as an isomer of gallocatechin/melacacidin. Compound **9** was assigned as procyanidin B2. Procyanidin dimers were found in *Acacia catechu* [46]. Compounds **10**–**15** were tentatively assigned as (−)-epicatechin, quercetin diglucoside, epicatechin gallate, rutin, quercetin-3-galactoside/myricitrin and kaempferol, because these compounds were reported in either *A. implexa* [13] or other *Acacia* species [41]. Of these compounds, the assignment of compound **10** as (−)-epicatechin was also supported by matching the RT and *m*/*z* with a standard (RT 5.068 min and *m*/*z* 289.0724).

Similar to *A. implexa* bark, earlier eluting compounds **4** and **9–12** were prominent in the crude extract and the EtOAc fraction, and are possible contributors to the antioxidant activity shown in the aqueous assay (Figure 1B) as they all contain the ortho-diphenol group. Later eluting compounds **13**−**15** were found in the *n*-hexane or DCM fractions and may have contributed to the antioxidant activities in the lipid-based assays (Figure 2C,D). Compounds **13** and **14** contain the ortho-diphenol group, and although compound **15** does not, it has been reported to have potent antioxidant activity [47].

**Table 3:** Tentative assignment of compounds in *Acacia implexa* leaves (crude extract).

* Comp	* RT	* *m*/*z* Expt	* *m*/*z* Calc	* MF	* δ in ppm	Putatively Identified Compound	Reference
4	2.629	305.0669	305.0667	C_15_H_14_O_7_	0.73	isomer of gallocatechin/melacacidin	[13,41]
9	4.679	577.1349	577.1351	C_30_H_26_O_12_	0.43	procyanidin B2	[48]
10	5.087	289.0721	289.0718	C_15_H_14_O_6_	1.17	(−)-epicatechin	* ST, [13,41]
11	6. 652	625.1397	625.1410	C_27_H_30_O_17_	2.11	quercetin diglucoside	[41]
12	7.093	441.0819	441.0827	C_22_H_18_O_10_	1.86	epicatechin gallate	[13,41]
13	7.360	609.1459	609.1461	C_27_H_30_O_16_	0.34	rutin	[41]
14	7.460	463.0887	463.0882	C_21_H_20_O_12_	1.08	quercetin-3-galactoside/myricitrin	[41]
15	9.175	285.0415	285.0405	C_15_H_10_O_6_	3.63	kaempferol	[41]

* Comp = compound, * RT = retention time, * *m*/*z* = mass to charge ratio, * expt. = experimental, * calc = calculated, * MF = molecular formula, * δ in ppm = error (ppm),* ST = standards.

#### 2.4.3. *Eucalyptus rossii* Leaves

In Table 4, compound **1** was assigned as gallic acid, which was also found in *A. implexa* bark. Compound **16** was assigned as (+)-catechin by matching with a standard (RT = 2.928 min and *m*/*z* = 289.0718). In addition, the assignment was supported by the review of Vuong et al. [38], in which the compound was reported to be present in other species of *Eucalyptus*. Similarly, compounds **17**–**20**, **22** and **23** were tentatively assigned as ellagic acid, cypellocarpin B, quercetin-3-glucoside, quercitrin, 5-hydroxy-7,4′-dimethoxy flavone and sideroxylin on the basis of Vuong et al. [38], where the compounds were reported in other species of *Eucalyptus.* Compound **21** was putatively annotated as 3-methyl ellagic acid, which was previously reported in *Eucalyptus cypellocarpa* [49]. Compound **24** was putatively annotated as eucalyptin on the basis of identification and characterisation in various species of *Eucalyptus* [50].

In *E. rossii*, compounds **1** and **16**–**22** were prominent in the crude extract and EtOAc fraction, whereas compounds **23** and **24** featured in the *n*-hexane or DCM fractions. Most of compounds **1**, **16**–**22** contained the ortho-diphenol group and are therefore likely to contribute to the strong antioxidant activity of the crude extract and EtOAc fraction in the aqueous ABTS assay (in Figure 1C). As far as the authors are aware, there is only one report [51] on the antioxidant activity of **23**, which was found to be not significant in an aqueous-based test. There are no reports on the antioxidant activity of **23** in the lipid-based assay, nor for **24** in any antioxidant tests. It is possible that **23** and **24** are the compounds responsible for inhibiting lipid oxidation (Figure 2E,F) as some compounds lacking ortho-diphenol groups are inactive in an aqueous environment, but active in a non-aqueous one [52].

**Table 4:** Tentative assignment of compounds in *Eucalyptus rossii* leaves (crude extract).

* Comp	* RT	* *m*/*z* Expt	* *m*/*z* Calc	* MF	* δ in ppm	Tentatively Identified Compound	Reference
1	0.730	169.0146	169.0142	C_7_H_6_O_5_	2.08	gallic acid	* ST, [38]
16	2.961	289.0720	289.0718	C_15_H_14_O_6_	0.82	(+)-catechin	* ST [38]
17	7.208	300.9994	300.9990	C_14_H_6_O_8_	1.38	ellagic acid	[38]
18	7.250	537.1978	537.1978	C_26_H_34_O_12_	0.09	cypellocarpin B	[38]
19	7.350	463.0876	463.0882	C_21_H_20_O_12_	1.29	quercetin-glucoside	[38]
20	7.974	447.0920	447.0933	C_21_H_20_O_11_	2.87	quercitrin	[38]
21	8.274	315.0150	315.0146	C_15_H_8_O_8_	1.14	3-methyl ellagic acid	[49]
22	12.613	297.0772	297. 0768	C_17_H_14_O_5_	1.18	5-hydroxy-7,4′-dimethoxy flavone	[38]
23	12.788	311.0927	311.0925	C_18_H_16_O_5_	0.65	sideroxylin	[38]
24	13.612	325.1846	325.1081	C_19_H_18_O_5_	0.11	eucalyptin	[50]

* Comp = compound, * RT = retention time, * *m*/*z* = mass to charge ratio, * expt = experimental, * calc = calculated, * MF = molecular formula, * δ in ppm = error (ppm), * ST = standards.

#### 2.4.4. *Exocarpos cupressiformis* Leaves

In Table 5, compounds **25**–**28** were putatively annotated as kaempferol-3-rhamnobioside, dihydrokaempferol-7-rhamnoside, kaempferol-7-rhamnoside and quercetin-3-rhamnobioside based on the identification and characterisation of these compounds in *E. cupressiformis* [15]. Compound **16** was assigned as (+)-catechin, and compound **20** was assigned as quercitrin, which is the rhamnoside of quercetin. As quercetin-3-rhamnobioside **28** was reported in this species of *Exocarpos*, it is possible that another glycoside, **20**, is also present.

Compounds **16**, **20** and **25**–**28** were found in the crude extract and all fractions, although amounts varied among the fractions. As has been discussed above, (+)-catechin (**16**), kaempferol (**15**), quercetin and their derivatives are recognised as having high antioxidant activity. (+)-Catechin (**16**) is the most polar of these compounds and is perhaps responsible for the high antioxidant activity of the EtOAc and EtOH fractions in the ABTS test (Figure 1D). Compounds **25** and **28** are the least polar and may be responsible for the exceptional antioxidant activity of the *n*-hexane and DCM fractions in the TBARS assay (Figure 2H).

Overall, the UHPLC-MS results show that the crude extract and fractions contain a wide variety of compounds of different polarities. The *n*-hexane and DCM fractions were found to contain compounds of lower polarity, which nonetheless were able to act as effective antioxidants in the lipid-based tests. Such compounds may not have been considered if only the ABTS assay was used for the screening of activity.

**Table 5:** Tentative assignment of compounds in *Exocarpos cupressiformis* leaves (crude extract).

* Comp	* RT	* *m*/*z* Expt	* *m*/*z* Calc	* MF	* δ in ppm	Tentatively Identified Compound	Reference
25	2.752	577.1352	577.1563	C_27_H_30_O_14_	0.16	kaempferol-3-rhamnobioside	[15]
16	2.936	289.0722	289.0707	C_15_H_12_O_6_	0.06	(+)-catechin	* ST standard
26	7. 432	433.1137	433.1140	C_21_H_22_O_10_	0.74	dihydro-kaempferol-7-rhamnoside	[15]
20	7.974	447.0929	447.0933	C_21_H_20_O_11_	0.86	quercitrin	[38]
27	8.523	431.0986	431.0984	C_21_H_20_O_10_	0.53	kaempferol-7-rhamnoside	[15]
28	10.488	593.1292	593.1512	C_27_H_30_O_15_	0.15	quercetin-3-rhamnobioside	[15]

* Comp = compound, * RT = retention time, * *m*/*z* = mass to charge ratio, * expt = experimental, * calc = calculated, * MF = molecular formula, * δ in ppm = error (ppm), * ST = standards.

In conclusion, this is the first report on the antioxidant activity testing and putative annotation for these three Australian medicinal plant species. The crude extracts showed good radical scavenging ability (ABTS test) and inhibition of peroxide formation (FTC assay). Crude extracts of AIB and EXOC were the most effective in inhibiting TBARS formation. EtOAc and EtOH fractions were consistently effective antioxidants in the ABTS test, congruent with this aqueous test favouring polar antioxidants. Conversely the *n*-hexane and DCM fractions typically showed good antioxidant activity in the lipid-based assay, especially inhibition of TBARS formation. The latter finding demonstrates the importance of multi-dimensional antioxidant testing, as the *n*-hexane and DCM fractions would not have undergone further testing based on the ABTS results. As this is often the case in the literature [1], it is important that there is a lipid-based assay suitable for high-throughput screening, as presented here. The results from this study, i.e., that extracts of AIB, AIL, EUR and EXOC have significant antioxidant activity, should lead to further studies on the phytochemistry of these plants, since there has been little work since preliminary reports in the 1960s.

## 3. Materials and Methods

### 3.1. Chemicals and Reagents

Linoleic acid (>99%) was purchased from Nu-Chek Prep Inc., Elysian, MN, USA. (±)-6-Hydroxy-2,5,7,8-tetramethylchromane-2-carboxylic acid (Trolox) (97%), gallic acid (97.5%), (+)-catechin hydrate (>98%), (−)-epicatechin (>90%), caffeic acid (>98%), α-tocopherol (>96%), quercetin (>95%), trichloroacetic acid (TCA) (>99%), thiobarbituric acid (TBA) (>98%), ammonium thiocyanate (>97.5%), polyoxyethylene sorbitan monolaurate (Tween 20) (97%), Folin–Ciocalteu reagent (≥97%), caffeine (≥98%), formic acid (~98%), ABTS (≥98%) and iron(II) chloride (98%) were obtained from Sigma-Aldrich (St Louis, USA). L-ascorbic acid (>99%) was purchased from Sigma-Aldrich (Beijing, China), copper(II) chloride dihydrate (>99%), hydrogen peroxide (30% *v*/*v*) and anhydrous sodium dihydrogen orthophosphate (sodium phosphate monobasic) (99%) from Chem-Supply (Adelaide, Australia), absolute ethanol (99.97%) and acetonitrile (≥99.9%) from VWR Internationals (Paris, France), methanol (99.99%) from Merck Chemicals (Darmstadt, Germany), nitric acid (70%, *v*/*v*) from Ajax FineChem Pty Ltd. (Auckland, New Zealand), potassium persulfate (99%) and hydrochloric acid (32%, *v*/*v*) from Ajax Finechem Pty Ltd. (Sydney, Australia), chloroform (>99%, HPLC grade) from Fisher Chemical (Loughborough, UK), cobalt(II) chloride hexahydrate (>98%) from M & B Laboratory Chemicals (London, UK) and anhydrous di-sodium hydrogen phosphate (sodium phosphate dibasic) (99.7%) from VWR International BVBA (Leuven, Belgium). Dichloromethane (DCM) (≥99.5%) and anhydrous sodium sulfate (≥95%) were obtained from Fisher Scientific (Loughborough, UK), and the ethyl acetate (EtOAc) (99.8%) was from Sharlau, S.L. (Barcelona, Spain). Anhydrous sodium carbonate (99.5%) was from Biolab (Adelaide, Australia). Ultra-pure water (Edwards Instruments Co, Sydney, Australia) was used throughout, and glassware was soaked overnight in 10% (*v*/*v*) aqueous nitric acid (prepared from 70% *v*/*v*, RCI, Labscan Ltd., Bangkok, Thailand).

### 3.2. Plant Species and Materials

Australian native plants, *A. implexa* (bark and leaves *) (AIB and AIL), *E. rossii* (leaves) (EUR) and *E. cupressiformis* (leaves *) EXOC were chosen for screening antioxidant activity. (* The photosynthetic units of *A. implexa* are phyllodes (flattened photosynthetic petioles), not leaves, while the photosynthetic organs of *E. cupressiformis* are cladodes (photosynthetic stems), with tiny scales leaves; in both cases, the term “leaves” is used throughout this paper, rather than potentially unfamiliar technical terms of “phyllode” and “cladode”.) Before collecting the plant materials, a scientific license (SL 101521, dated 2 April 2015) was approved from the office of Environment & Heritage, NSW National Parks & Wildlife Service. All plants were mature trees, and samples were taken from at least five individuals spread over an area of approximately 100 m^2^. The plant materials of AI were collected on 12 March 2015 from Rocky Hill, Wagga Wagga, NSW, and consisted of outer dead bark and mature leaves. The trees were in flower, but no buds or fruit were present. Mature leaves from EUR were collected on 1 May 2015 from Livingstone National Park, Big Springs, NSW. The trees were in flower and small fruit were also present. Mature leaves from EXOC were collected on 15 May 2015 from the Blowering Dam access road, NSW. The trees were in flower, but no buds or fruit were present. The three places are located within Wiradjuri country. Samples of the plant species were identified, vouched (AI-NSW 981212, EUR-NSW 981273 and EXOC-NSW 981279) and archived at the National Herbarium of New South Wales.

### 3.3. Preparation and Fractionation of Plant Extracts

Plant material was collected into large zip-lock plastic bags, then taken directly to the laboratory for further treatment. The amounts of plant materials collected were 2 kg AIB, 1 kg AIL, 2 kg EUR and 3 kg EXOC, which were separated from foreign matter and air-dried at ambient temperature until constant weight was attained. The plant materials were then cut into smaller pieces and ground into fine powder using a blender (Breville, Sydney, Australia). Two suspensions, one of 1000 g of ground AIB in 1700 mL 70% (*v*/*v*) aqueous ethanol, and a second suspension of 500 g each of AIL, EUR and EXOC in 1600 mL in the same solvent, were prepared in conical flasks, covered by aluminium foil and shaken for 15 h in a shaker incubator at 150 rpm at room temperature. The plant materials were centrifuged (Eppendorf centrifuge 5810) at 4000 rpm for 20 min using 400 mL wide-mouth plastic bottles (Eppendorf, Hamburg, Germany). The residue was extracted twice with the same solvent. For each extraction, the residue of AIB was suspended in 1000 mL, and the residue of EUR or EXOC in 600 mL, of 70% (*v*/*v*) aqueous ethanol. The three aqueous-ethanolic extracts were combined, and ethanol was removed by rotary evaporation (Buchi, Adelaide, Australia) under vacuum at 37 °C. The aqueous residue was freeze dried (76 h) using an Alpha 2–4 LDplus freeze dryer (John Morris Scientific, Sydney, Australia) to afford the crude extracts.

A 50 g sample of the crude extract was suspended in 200 mL water, placed in a separatory funnel and successively partitioned with *n*-hexane (3 × 100 mL), dichloromethane (DCM) (3 × 100 mL) and ethyl acetate (EtOAc) (3 × 100 mL). For each partition, the solvent was dried with anhydrous sodium sulfate (2 g) and solvent was removed by rotary evaporation under vacuum at 37 °C to afford the *n*-hexane, DCM and EtOAc fractions. The remaining aqueous layer was discarded, and the solid residue was extracted with absolute ethanol (EtOH) (3 × 100 mL). The EtOH fraction was obtained by removing solvent by rotary evaporation, as above. For convenience, the crude extract and *n*-hexane, DCM, EtOAc and EtOH fractions were denoted as, for *A. implexa* bark, AIB crude, AIB H, AIB DCM, AIB EtOAc, AIB EtOH; for *A. implexa* leaves, AIL crude, AIL H, AIL DCM, AIL EtOAc, AIL EtOH; for *E. rossii*, EUR crude, EUR H, EUR DCM, EUR EtOAc, EUR EtOH; and for *E. cupressiformis*, EXOC crude, EXOC H, EXOC DCM, EXOC EtOAc and EXOC EtOH. Extraction yields were calculated as follows:

Crude yield: (mass of crude extract/mass of dried plant material) × 100%

Fraction yield: (mass of fraction/mass of crude extract) × 100%

### 3.4. Determination of Total Phenolic Content

Total phenolic content of crude extracts and fractions were determined using the Folin–Ciocalteu test in accordance with the method of Qawasmeh et al. [53]. An aliquot of 100 µL crude extract or fraction (dissolved in absolute EtOH, 1 mg/mL) was added to a 10 mL volumetric flask containing 5 mL water. Five hundred microlitres of Folin–Ciocalteu reagent were added, and after 1 min, 1.5 mL 20% (*w*/*v*) aqueous sodium carbonate was added. The mixture was shaken well, the total volume was made up to 10 mL with water, mixed thoroughly and incubated for 1 h at ambient temperature. The absorbance was measured at 760 nm (Cary 4000 spectrophotometer, Agilent Technologies, Melbourne, Australia). Results were expressed as milligrams of gallic acid equivalent per gram dry weight of extract or fraction, based on a seven-point calibration curve—0.0, 0.1, 0.2, 0.3, 0.4, 0.5 and 0.6 mg/mL gallic acid in 70% (*v*/*v*) aqueous ethanol (*r*^2^ = 0.999).

### 3.5. Free Radical Scavenging 2,2′-Azino-bis (3-ethylbenzothiazoline-6-sulfonic acid) (ABTS) Assay

The radical scavenging activity of the crude extracts and fractions was determined following the method of Qawasmeh et al. [53]. A stock solution of ABTS radical (ABTS^•+^) was prepared by adding aqueous ABTS solution (7.0 mM) and aqueous potassium persulfate (2.45 mM) in a stoppered Schott bottle wrapped in aluminium foil. The bottle was incubated in darkness overnight (15 h) at room temperature. The working solution of ABTS^•+^ was prepared by diluting a 1 mL aliquot stock solution in 37 mL water to a final absorbance of 0.720 ± 0.003 at 734 nm. Then, 50 µL of crude extract or fraction (dissolved in absolute EtOH, 1 mg/mL) was added to 3 mL of working solution of ABTS^•+^ in a 1 cm plastic cuvette. The cuvette was covered, shaken well and incubated at room temperature for 30 min. The absorbance at 734 nm was measured and the scavenging activities of the crude extracts and fractions were calculated as a percentage:

% scavenging = (A_control_ − A_sample_)/A_control_ × 100% (1)

where A_control_ is the absorbance when 50 μL of ethanol was added instead of crude extract or fraction.

### 3.6. Lipid-Based Ferric Thiocyanate (FTC) and Thiobarbituric Acid Reactive Substances (TBARS) Assays

#### 3.6.1. Oxidation of a Linoleic Acid (LA) Nano-Emulsion

A LA nano-emulsion was prepared following the method of Ghani et al. [6]. Then, 2.5 mL homogenised (nano droplet size < 250 nm and polydispersity index < 0.7) 0.02 M LA emulsion (pH 7), 0.5 mL (1 mg/mL) crude extract or fraction and 2 mL 0.2 M aqueous phosphate buffer solution (PBS) (pH 7) were mixed together in a 50 mL centrifuge tube. The negative control was 0.5 mL PBS, and the positive control 0.5 mL Trolox (1 mg/mL in 50:50 methanol:water *v*/*v*). After the mixture had been mixed thoroughly, it was oxidised at 50 °C for 5 h in a water bath. Two aliquots, 100 µL, were taken from the oxidation mixture for the measurement of peroxides and TBARS, as discussed below.

#### 3.6.2. Ferric Thiocyanate (FTC) Assay

Peroxides were measured following the FTC method of Ghani et al. [6]. An aqueous solution of ethanol (4.7 mL, 75% *v*/*v*), aqueous ammonium thiocyanate (0.1 mL, 30%), the oxidation sample (100 µL) and iron(II) chloride (0.1 mL, 0.02 M in 3.2% hydrochloric acid) were sequentially added in a 10 mL conical flask and the peroxides were determined at 500 nm against a blank containing all reagents except LA. The antioxidant activity (% inhibition of peroxide formation) was calculated:

% inhibition of peroxide formation = (A_control_ − A_sample_)/A_control_ × 100% (2)

#### 3.6.3. Thiobarbituric Acid Reactive Substances (TBARS) Assay

TBARS were measured following the method of Ghani et al. [6]. A 100 µL aliquot of oxidation sample was added to 2 mL TBA-TCA solution (20 mM TBA in 15% (*v*/*v*) TCA) in a 10 mL centrifuge tube and heated for 15 min in a boiling water bath. The sample was cooled to room temperature and the absorbance of the aqueous layer was measured at 532 nm against a blank using all reagents except LA. The antioxidant activity (% inhibition of TBARS formation) was calculated using equation (2).

### 3.7. Ultra High Pressure Liquid Chromatography Quadrupole Time-of-Flight Mass Spectrometry (UHPLC-qTOF-MS) Analysis

#### 3.7.1. Crude Extracts and Fractions Sample Preparation

A 1 mg/mL solution for each crude extract was prepared in 70% (*v*/*v*) aqueous ethanol, while the DCM, EtOAc and EtOH fractions were prepared in 50% (*v*/*v*) aqueous MeOH. The *n*-hexane fraction was dissolved in absolute ethanol, also at 1 mg/mL concentration. The samples were filtered through 0.22 µm syringe filters before injecting in the UHPLC-DAD-qTOF-MS.

#### 3.7.2. Instrumentation

The UHPLC-qTOF-MS system comprised a quaternary pump (1260 series), a diode-array detector (DAD) (1200 series), a 6530 accurate mass analyser, and an autosampler (1200 series) all from Agilent Technologies (Santa Clara, USA). Separation was performed with a Poroshell 120 SB-C-18 column 2.1 mm × 100 mm, 2.7 µm particle size (Agilent Technologies, Santa Clara, USA), with the column compartment at 40 °C and an injection volume of 2 µL. The flow rate was 0.6 mL/min and maximum column pressure was set at 550 bar. Gradient elution was performed with 0.2% (*v*/*v*) formic acid in water (solvent A) and 0.2% (*v*/*v*) formic acid in acetonitrile (solvent B) as follows: 0–1 min, 5% B; 1–4 min increase to 10% B; 4–10 min increase to 40% B; 10–13 min increase to 100% B; 13–16 min hold at 100% B; 16–21 min decrease to 5% B; 21–25 min hold at 5% B. The DAD was set at 280 nm and 380 nm. Mass spectra were collected under the following conditions: mass range (*m*/*z*), 100–1600; nozzle voltage (V), 1000; fragmentor voltage (V), 150; gas temperature, 300 °C; gas flow, 10 L/min; nebuliser psig, 45; in the negative ionisation mode. UHPLC-qTOF-MS data were analysed using MassHunter Workstation, Qualitative Analysis Software (version B. 07. 00, 2014) (Agilent Technologies, USA).

### 3.8. Statistical Analysis

All samples were prepared in triplicate and results are expressed as mean ± standard deviation. The statistical significance of differences was determined by one-way ANOVA and Tukey’s honest significant difference (HSD) post hoc test using the SPSS software, version 20 (SPSS Inc., Chicago, USA). Results were considered to be statistically significant at *p* < 0.05.

## Figures and Tables

**Table 1:** Extraction yield and total phenolic content of crude extracts and fractions of crude extracts. Different letters in the same column show significant difference at *p* < 0. 05 (Tukey’s honest significant difference (HSD) post hoc test). Data are expressed as mean ± S.D.

Plant Species	Crude Extracts and Fractions	Extraction Yield (% Yield)	Total Phenolic Content (mg GAE/g Dry Extract)
*A. implexa* bark (AIB)	Crude (AIB)	11	439.5 ± 19.7 ^c^
	AIB H *	0.08	53.2 ± 2.7 ^h^
	AIB DCM *	0.5	149.1 ± 2.1 ^f^
	AIB EtOAc *	3	328.5 ± 11.8 ^d^
	AIB EtOH *	34	408.8 ± 21.6 ^c^
*A. implexa* leaves (AIL)	Crude (AIL)	28	195.2 ± 10.1 ^e^
	AIL H	0.5	42.4 ± 2.1 ^h^
	AIL DCM	0.3	98.6 ± 5.0 ^g^
	AIL EtOAc	3	583.2 ± 32.5 ^a^
	AIL EtOH	1	118.7 ± 8.5 ^g^
*E. rossii* (EUR) (leaves)	Crude (EUR)	30	419.2 ± 15.7 ^c^
	EUR H	3	106.4 ± 6.2 ^g^
	EUR DCM	15	159.9 ± 4.4 ^f^
	EUR EtOAc	11	530.5 ± 30.2 ^b^
	EUR EtOH	0.8	439.7 ±21.1 ^c^
*E. cupressiformis* (EXOC) (leaves)	Crude (EXOC)	28	437.6 ± 15.5 ^c^
	EXOC H	1	59.6 ±4.1 ^h^
	EXOC DCM	5	185.8 ± 21.5 ^e^
	EXOC EtOAc	30	413.8 ± 20.2 ^c^
	EXOC EtOH	3	417.5 ± 6.2 ^c^

* H = *n*-hexane, DCM = dichloromethane, EtOAc = ethyl acetate, EtOH = ethanol fractions, GAE = gallic acid equivalents.

## Data Availability

The data supporting this study’s findings are available upon request to the corresponding author.
