# Correction: Ghani et al. Multi-Dimensional Antioxidant Screening of Selected Australian Native Plants and Putative Annotation of Active Compounds. *Molecules* 2023, *28*, 3106

**DOI:** 10.3390/molecules29040893

**Published:** 2024-02-18

**Authors:** Md. Ahsan Ghani, Celia Barril, Danny R. Bedgood, Geoffrey E. Burrows, Danielle Ryan, Paul D. Prenzler

**Affiliations:** 1 School of Agricultural, Environmental and Veterinary Sciences, Charles Sturt University, Wagga Wagga, NSW 2650, Australia; gahsan23@yahoo.com (M.A.G.); cbarril@csu.edu.au (C.B.); dbedgood@csu.edu.au (D.R.B.J.); gburrows@csu.edu.au (G.E.B.); dryan@csu.edu.au (D.R.); 2 The Gulbali Institute, Charles Sturt University, Wagga Wagga, NSW 2650, Australia

In the original publication [1], the author list was incomplete due to miscommunication. The correct list of authors is provided below.

**Original:**

Md Ahsan Ghani ^1^, Celia Barril ^1^, Danny R. Bedgood, Jr. ^1^, Geoffrey E. Burrows ^1^ and Paul D. Prenzler ^1,2,^*

^1^ School of Agricultural, Environmental and Veterinary Sciences, Charles Sturt University, Wagga Wagga, NSW 2650, Australia; mghani@csu.edu.au (M.A.G.); cbarril@csu.edu.au (C.B.); dbedgood@csu.edu.au (D.R.B.J.); gburrows@csu.edu.au (G.E.B.)

^2^ The Gulbali Institute, Charles Sturt University, Wagga Wagga, NSW 2650, Australia

* Correspondence: pprenzler@csu.edu.au

**This should be replaced with the following:**

Md. Ahsan Ghani ^1^, Celia Barril ^1^, Danny R. Bedgood, Jr. ^1^, Geoffrey E. Burrows ^1^, Danielle Ryan ^1^ and Paul D. Prenzler ^1,2,^*

^1^ School of Agricultural, Environmental and Veterinary Sciences, Charles Sturt University, Wagga Wagga, NSW 2650, Australia; gahsan23@yahoo.com (M.A.G.); cbarril@csu.edu.au (C.B.); dbedgood@csu.edu.au (D.R.B.J.); gburrows@csu.edu.au (G.E.B.); dryan@csu.edu.au (D.R.)

^2^ The Gulbali Institute, Charles Sturt University, Wagga Wagga, NSW 2650, Australia

* Correspondence: pprenzler@csu.edu.au

All authors agree with the content of this correction and wish to apologise for any inconvenience to the readers resulting from this error.
